# Integrin Targeting and Beyond: Enhancing Cancer Treatment with Dual-Targeting RGD (Arginine–Glycine–Aspartate) Strategies

**DOI:** 10.3390/ph17111556

**Published:** 2024-11-20

**Authors:** Bojana Bogdanović, Daniel Fagret, Catherine Ghezzi, Christopher Montemagno

**Affiliations:** 1 INSERM, CHU Grenoble Alpes, Laboratory of Bioclinical Radiopharmaceutics, University Grenoble Alpes, 38000 Grenoble, France; bojana.bogdanovic@univ-grenoble-alpes.fr (B.B.); daniel.fagret@univ-grenoble-alpes.fr (D.F.); catherine.ghezzi@univ-grenoble-alpes.fr (C.G.); 2 Biomedical Department, Centre Scientifique de Monaco, 98000 Monaco, Monaco

**Keywords:** RGD-binding integrin, αvβ3, dual targeting, theranostic, solid tumors, PET imaging

## Abstract

Integrins, an important superfamily of cell adhesion receptors, play an essential role in cancer progression, metastasis, and angiogenesis, establishing them as prime targets for both diagnostic and therapeutic applications. Despite their significant potential, integrin-targeted therapies have faced substantial challenges in clinical trials, including variable efficacy and unmet high expectations. Nevertheless, the consistent expression of integrins on tumor and stromal cells underscores their ongoing relevance and potential. Traditional RGD-based imaging and therapeutic agents have faced limitations, such as inconsistent target expression and rapid systemic clearance, which have reduced their effectiveness. To overcome these challenges, recent research has focused on advancing RGD-based strategies and exploring innovative solutions. This review offers a thorough analysis of the latest developments in the RGD–integrin field, with a particular focus on addressing previous limitations. It delves into new dual-targeting approaches and cutting-edge RGD-based agents designed to improve both tumor diagnosis and therapeutic outcomes. By examining these advancements, this review illuminates new pathways for enhancing the specificity and efficacy of integrin-targeted therapies, paving the way for more effective cancer diagnosis and treatment strategies.

## 1. Introduction

Cancer remains a critical global health challenge, with an estimated 19.3 million new cases and 10 million deaths reported worldwide in 2020, according to GLOBOCAN. The disease now accounts for nearly 1 in 6 deaths globally, making it the second leading cause of mortality [1]. Despite progress in early detection and treatments, the global burden of cancer continues to rise, with projections indicating a 47% increase in new cases by 2040, reaching over 28 million annually [1,2,3,4]. This surge, driven by population aging and growth, alongside changing risk factors such as lifestyle and environmental exposures, underscores the escalating challenge faced by healthcare systems worldwide.

Despite progress in early detection and conventional treatments like chemotherapy and radiation, the burden of cancer continues to rise, with projections exceeding 35 million new cases by 2050 [2]. This stark reality underscores the urgent need for more precise and effective diagnostic and therapeutic approaches.

Historically, cancer treatment was limited primarily to surgery, which aimed to remove tumors. However, as our understanding of cancer biology has advanced, so too have the available therapies. Today, cancer treatment options include surgery, chemotherapy, radiation therapy, immunotherapy, and targeted therapies. Each of these approaches can be tailored based on the type of cancer, its stage, and the individual patient’s needs [5]. Personalized and precision medicine are transforming cancer care by offering more targeted and efficient strategies [6]. This approach delves into the molecular foundations of cancer, enabling the identification of unique patient-specific targets, such as overexpressed receptors and tumor-associated proteins. For instance, agents based on peptides, antibodies, and nanoparticles offer highly specific targeting in both preclinical cancer models and patients’ disease states through targeted imaging, while also aiming to eliminate cancer cells with therapies that minimize harm to healthy tissue [7]. Despite these promising advancements, current targeting strategies face challenges such as tumor heterogeneity, drug resistance, and non-specific uptake by healthy tissues [8]. These hurdles can compromise both diagnostic accuracy and therapeutic effectiveness, highlighting the need for ongoing research and innovation in this dynamic field.

Integrins, particularly αvβ3, have emerged as prominent targets in precision oncology due to their involvement in critical processes of cancer progression, including angiogenesis, invasion, and metastasis [9]. A broad array of αvβ3-targeting agents has been developed over the years, with many incorporating the well-characterized RGD (arginine–glycine–aspartate) motif, known for its high affinity binding to this integrin [10,11]. These agents range from small peptides to monoclonal antibodies and nanoparticle conjugates. However, despite promising preclinical results, the clinical translation of RGD-based integrin-targeting agents has faced significant challenges, including inadequate tumor selectivity, heterogeneous αvβ3 expression, and the rapid clearance of small RGD peptides [11,12,13,14].

To address these limitations, dual-targeting strategies are being actively investigated. By simultaneously engaging two distinct receptors or proteins on cancer cells, in the tumor microenvironment or on immune cells, dual-targeting agents offer improved tissue uptake and enhanced pharmacokinetics compared to their monovalent counterparts [15]. Combining αvβ3 integrin targeting via the RGD motif with additional tumor-specific markers has demonstrated potential for enhancing the precision and efficacy of these agents [16,17]. This review explores the evolving landscape of dual-targeting strategies, focusing on RGD-based integrin-targeting approaches involving αvβ3, and their potential to provide more powerful tools for cancer diagnosis and therapy.

## 2. Integrins—Unveiling Tumor Dynamics

### 2.1. Fundamentals of Integrin Biology

Integrins are a large family of transmembrane adhesion receptors that play a vital role in cell communication and signaling. These receptors are composed of heterodimeric complexes formed by α and β subunits, resulting in 24 unique receptors in humans [18,19,20]. Each subunit is a type 1 transmembrane protein. These proteins have a large extracellular domain for ligand binding and a smaller transmembrane and intracellular region that participates in cell signaling [21].

Integrins can bind a wide variety of ligands, including insoluble extracellular matrix (ECM) proteins (e.g., fibronectin, laminin, collagen), matricellular proteins (e.g., Cyr61, CTGF, NOV), cell surface proteins (e.g., ICAMs, VCAM-1), and soluble factors (e.g., fibrinogen, complement proteins, VEGF, FGF2, TGFβ) [22]. Integrins exhibit promiscuous binding, where a single integrin can bind multiple ligands, and redundancy, where different integrins can bind the same ligand [22]. This flexibility enables integrins to support diverse cellular functions in dynamic environments, allowing cells to respond in multiple ways to the same ECM proteins. Integrins are grouped into four receptor classes based on their ligand-binding specificities: collagen receptors (recognized by α1β1, α2β1, α10β1 and α11β1), laminin receptors (recognized by α3β1, α6β1, α6β4 and α7β1), leukocyte-specific (recognized by α9β1, α4β1, α4β7, αEβ7, αLβ2, αMβ2, αXβ2 and αDβ2), and Arg-Gly-Asp (RGD) [19,23,24]. The RGD motif, recognized by eight integrins (α5β1, α8β1, αvβ1, αvβ3, αvβ5, αvβ6, αvβ8, and αIIbβ3), is notably found in several molecules, including ECM proteins like fibronectin and vitronectin [25].

Integrins have two main functions: mediating cellular or ECM adhesion and facilitating signal transduction through outside–in and inside–out signaling. Outside–in signaling transmits signals from ECM ligands to elicit specific cellular responses, while inside–out signaling triggers conformational changes to adjust ligand affinity [26,27]. Integrins exhibit dynamic conformational flexibility, switching between active (high affinity) and inactive (low affinity) states [21,28]. The active state is regulated by intracellular adaptor proteins talin and kindlin binding to the β-subunit cytoplasmic tail and is stabilized by ligand binding at the extracellular domain [29]. To ensure firm adhesion, integrins must cluster into adhesion complexes that link to the cytoskeleton.

Upon activation, integrins recruit several kinases such as SRC family kinases (SFKs), focal adhesion kinase (FAK), and integrin-linked kinase (ILK), which activate downstream pathways, including phosphoinositide 3-kinase (PI3K)/protein kinase B (PKB/AKT), mitogen-activated protein kinase (MEK)/extracellular signal-regulated kinase (ERK), and yes-associated protein (YAP)/transcriptional coactivator with PDZ-binding motif (TAZ) [26,30]. These signaling cascades affect various aspects of cell behavior, including survival, proliferation, metabolism, differentiation, shape, and motility [31]. While integrins are vital for physiological processes like embryogenesis, immune response, wound healing, and angiogenesis, they are also implicated in pathological conditions such as cancer, cardiovascular diseases, and inflammation [32,33,34,35,36].

### 2.2. RGD-Binding Integrins in Cancer Progression

Dysregulation of integrins is a defining feature of numerous malignancies, with alterations in their expression frequently observed to promote tumor growth, survival, and metastasis [35]. Among them, integrins that recognize the RGD motifs, such as αvβ3, αvβ5, αvβ6, and α5β1, are critically involved in cancer progression [37,38,39,40]. These integrins facilitate essential oncogenic processes, including tumor cell adhesion, proliferation, migration, invasion, and contributing to neovascularization, immune evasion, and resistance to therapies [9].

Integrins-αvβ3, -αvβ6, and -α5β1, which are typically expressed at low levels in normal epithelia tissues, are frequently upregulated in tumors [35]. A key function of these integrins is the regulation of neovascularization. This process involves the formation of new blood vessels that supply essential nutrients and oxygen to the tumor, thereby supporting its growth [41,42]. Notably, integrin-αvβ3, which is expressed at minimal levels in quiescent endothelial cells, is significantly upregulated during tumor angiogenesis, where it plays a pivotal role in neovascularization [33,43]. Recent research has expanded our understanding of integrins beyond their conventional roles, implicating them in processes such as epithelial-to-mesenchymal transition (EMT), cancer stemness, and metabolic rewiring [44,45,46]. Moreover, integrins like αvβ6 and αvβ8 are involved in the activation of TGFβ, a key regulator of tumor immune evasion and suppression of anti-tumor immunity [47,48,49]. These findings suggest that integrins not only contribute to the structural aspects of tumor progression but also actively modulate the tumor microenvironment and immune landscape.

The role of RGD-recognizing integrins in metastasis is increasingly recognized as a critical area of investigation. Integrin-αvβ3, -αvβ5, and -αvβ6 have been implicated in promoting metastasis to distant organs, such as the lungs and bones. Additionally, αvβ3, αvβ5, and αvβ8 facilitate the transmigration of tumor cells cross the blood–brain barrier, enabling the establishment of brain metastasis [50,51,52,53,54,55]. Integrin-α5β1 has also been identified as a mediator of liver and bone metastasis through pathways involving c-Met, Src, and focal adhesion kinase (FAK) [56].

Emerging evidence also highlights the role of exosomes in mediating metastatic processes, with integrins being highly expressed on these vesicles [57]. For example, αvβ5-expressing exosomes target liver macrophages to enhance liver metastasis, while αvβ6-expressing exosomes from prostate cancer cells promote cell migration and metastasis in a paracrine manner. Similarly, αvβ3 on breast cancer-derived exosomes has been linked to an increased propensity for lung metastasis [51,58,59].

These insights underscore the pivotal role of integrins in cancer progression and underscore their potential as therapeutic targets in oncology. Consequently, significant efforts have been directed toward developing novel anticancer therapies that target integrins, with the aim of inhibiting tumor progression, preventing metastasis, and overcoming therapeutic resistance.

## 3. RGD Peptides in Focus—Bridging Cancer Diagnosis and Targeted Therapy

### 3.1. Targeting RGD-Recognizing Integrins for Cancer Diagnosis

The discovery of the integrin-binding RGD domain has driven the development of RGD-based peptides for cancer diagnosis and treatment, representing a significant advancement in oncology therapeutics and imaging strategies [11]. These peptides can directly inhibit integrins or serve as delivery for anticancer drugs and imaging agents, thereby enhancing treatment efficacy while minimizing off-target effects and damage to healthy tissues.

Imaging techniques have become invaluable tools in cancer diagnosis due to their non-invasive nature and ability to provide detailed insights into tumor biology [60]. By conjugating RGD peptides with radioactive or fluorescent probes, it becomes feasible to visualize and monitor tumors with high integrin expression, using advanced imaging modalities such as positron emission (PET) and single-photon emission tomography (SPECT) [61]. Numerous RGD radiotracers have shown promising results in preclinical and clinical studies across a range of cancers including ovarian, breast, lung, head and neck, non-small cell lung, and cervical cancers [62].

Among all RGD-binding integrins, αvβ3 has emerged as a prominent target, garnering extensive interest in the development of tumor-targeting radiotracers. For diagnostic imaging, gamma-emitting radionuclides such as the Technetium-99 m (^99m^Tc) are used in SPECT, while positron-emitting radionuclides such as Fluorine-18 (^18^F) and Gallium-68 (^68^Ga) are employed in PET [60,61,63]. Some RGD-derived tracers dedicated to imaging are summarized in Table 1.

For instance, ^18^F-Galacto-RGD PET has been evaluated to detect and visualize αvβ3 integrin expression in various cancers. In glioblastoma (GBM) (SUV range, 0.8–2.8; mean, 1.6 ± 0.5) and in head and neck squamous cell carcinoma (HNSCC) (SUV range, 2.2–5.8; mean, 3.4 ± 1.2), it allows for non-invasive monitoring of αvβ3 integrin expression [64,65]. Additionally, ^18^F-Galacto-RGD has been effective in visualizing bone metastases in prostate cancer patients [66].

Similarly, ^18^F-Alfatide has shown promise in identifying malignant lung tumors with high tumor-to-background ratios [67]. Additionally, all malignant lymph nodes in patients with lung tumors were successfully visualized on ^18^F-Alfatide PET/CT in patients, and the sensitivity, specificity, and accuracy were 100.0%, 94.9%, and 95.4%, respectively [68]. ^18^F-Alfatide has also been positively evaluated for αvβ3 imaging in GBM, with uptake levels correlating with tumor grade. It has also shown potential for assessing GBM response standard therapies [69,70].

^68^Ga-NOTA-PRGD2 PET/CT is another imaging tool that has been evaluated in multiple cancer types. In GBM, it has shown superior accuracy in visualizing αvβ3 integrin expression, assessing tumor grade, and differentiating between high-grade glioma and uncommon meningioma [71,72]. Studies conducted in lung malignancies demonstrated greater specificity for ^68^Ga-NOTA-PRGD2 than the current gold standard, ^18^F-FDG PET/CT, for the detection of lymph node metastasis with positive and negative predictive values of 90.0% (27/30) and 93.8% (121/129), respectively, whereas those of ^18^F-FDG PET/CT were 30.2% (29/96) and 90.5% (57/63), respectively [73]. Such results were also confirmed using ^68^Ga-DOTA-RGD2 PET/CT. ^68^Ga-DOTA-RGD2 enhances the visualization of angiogenesis in HNSCC and is effective in detecting primary and metastatic lymph nodes in breast cancer, surpassing ^18^F-FDG in specificity and accuracy for thyroid cancer detection [74,75]. Notably, in cases of chondrosarcoma, ^18^F-FDG showed limited uptake due to low mitotic activity. However, ^68^Ga-DOTA-RGD2 PET/CT showed significant uptake, suggesting a potential role for angiogenesis-targeted radiopharmaceuticals in refractory cases [76].

Among the recently developed RGD-based radiotracers, ^68^Ga-NODAGA-E[c(RGDyK)]2 has garnered significant attention due to its promising clinical performance. In a first-in-human phase I trial, ^68^Ga-NODAGA-E[c(RGDyK)]2 was safely used for imaging integrin αvβ3 in patients with breast cancer and in NEN (neuroendocrine neoplasms) patients, exhibiting low radiation exposure and favorable tumor monitoring [77]. In a phase II study, high tumor uptake was noted across all NEN grades, with a correlation between increased uptake and poorer prognosis [78]. High integrin α_v_β_3_ expression (defines as SUV_max_ > 5.25) had a hazard ratio of 2.11 and 6.95 for progression-free survival and overall survival, respectively (*p* = 0.01 for both). Additionally, this tracer was employed to assess RGD-binding integrin expression in thyroid cancer patients with negative radioiodine scintigraphy, revealing high expression in metastatic lesions, particularly in bone metastases [79]. Further research is warranted to validate its potential as a predictive tool for selecting patients suitable for integrin αvβ3-targeted therapies.

Finally, a phase 3 clinical trial of ^99m^Tc-3PRGD2 (NCT04233476) showed safety and efficacy for the diagnosis of lung cancer, potentially paving the way for its approval as a novel radiopharmaceutical for cancer diagnosis.

Beyond αvβ3, RGD radiotracers targeting other integrins have also been developed and evaluated in clinical translational studies. The ^68^Ga-labeled cyclic peptide ^68^Ga-cycratide, designed to target integrin αvβ6 via the RGDLATL sequence, exhibited enhanced serum stability and increased tumor uptake in comparison to the linear form, showing promise in detecting pancreatic neoplastic lesions and postoperative recurrence [80]. The tumor uptake of ^68^Ga-cycratide was significantly higher than that of ^68^Ga-linear-pep (2.15 ± 0.46 vs. 0.94 ± 0.58%ID/g; *p* < 0.05). The αvβ6-targeting peptide ^18^F-αvβ6-BP has shown effective tumor imaging, underscoring its clinical potential across various malignancies due to its ability to selectively target tumors with high αvβ6 expression [81]. Similarly, the tracer ^18^F-FP-R01-MG-F2 has yielded promising results in a pilot-phase PET/CT study, demonstrating safety and favorable radiation dosimetry in patients with pancreatic cancer, further supporting its potential as a diagnostic tool in αvβ6-positive tumors [82].

These advancements in RGD-based radiotracers are poised to enhance cancer detection and monitoring, providing novel insights into tumor biology and opening avenues for more targeted and effective therapies. Moreover, RGD peptides are being developed not only as diagnostic agents but also as therapeutic agents, underscoring their dual potential in oncology.

### 3.2. RGD-Based Peptides for Cancer Therapy

#### 3.2.1. Pharmacological Targeting of Integrins

A range of RGD-containing peptides have been developed to inhibit angiogenesis and tumorigenesis. Among these, Cilengitide (cyclo-Arg-Gly-Asp-DPhe-NMe-Val), a cyclic pentapeptide that blocks the RGD binding site, has emerged as a selective inhibitor of αvβ3 and αvβ5 integrins [83]. While preclinical studies demonstrated its efficacy in inhibiting angiogenesis and glioblastoma (GBM) growth, and early-phase clinical trials showed moderate anti-tumor effects, later-phase trials were less successful [84,85,86]. A phase II trial even suggested improved overall survival rates with Cilengitide [87]. However, subsequent larger-scale trials, including the randomized phase III CENTRIC and phase II CORE studies, failed to show significant improvements in overall survival with the addition of Cilengitide [12,13]. The limited success in these later trials may be attributed to the tumor microenvironment’s complexity, which can activate compensatory VEGF pathways and resistance mechanisms [88]. Nevertheless, in a retrospective study, high levels of αvβ3 were associated with anti-tumor response to Cilengitide, suggesting the potential need for patient stratification based on αvβ3 expression [89]. Furthermore, Cilengitide’s fast blood clearance underscores the necessity of alternative strategies, such as combining RGD peptides with other molecules, to improve integrin-targeting therapies.

#### 3.2.2. RGD-Based Peptides for Drug Delivery

RGD-based peptides are also increasingly explored for their ability to enhance the targeted delivery of therapeutics, including chemotherapy agents, peptides, and nucleic acids, via nanocarriers such as liposomes, nanoparticles, and micelles in pre-clinical studies [90]. RGD peptide-modified nanoparticles, such as solid lipid nanoparticles (SLNs) and pH-sensitive nanoparticles (RGD-NAMs), have demonstrated improved oral bioavailability and tumor targeting [91,92]. RGD-DOX-SLNs loaded with doxorubicin offer superior tumor inhibition and reduced side effects in breast cancer models, while RGD-NAMs enable controlled drug release through temperature- and pH-sensitive mechanisms [91,93]. Moreover, paclitaxel-loaded RGD nanoparticles have shown improved targeting and efficacy in lung cancer treatment, leading to significant tumor reduction and reduced systemic toxicity compared to free drugs [94]. The LCP-RGD nanoparticle, designed with a calcium phosphate core and RGD-modified components, provides an effective platform for co-delivering docetaxel (DTXL) and GRP78 siRNA. This nanoparticle demonstrates high drug and siRNA loading capacity, and enhanced anti-cancer effects, making it a promising tool for overcoming drug resistance in castration-resistant prostate cancer [95].

#### 3.2.3. RGD-Based Radiotracers for Targeted Radiotherapy

Certain RGD-based radiopharmaceuticals in nuclear medicine not only assist with diagnosis but also provide therapeutic benefits through targeted radiotherapy, especially in oncology, known as the ‘theranostic approach’ [96]. In these treatments, α- and β-particle-emitting radionuclides like Iodine-131 (^131^I), Lutetium-177 (^177^Lu), and Yttrium-90 (^90^Y) are preferred for their high linear energy transfer (LET) [60,61,63]. In preclinical studies, RGD radiotracers like ^177^Lu-3PRGD2 and RAFT-RGD labeled with ^67^/^64^Cu, ^177^Lu, or ^90^Y have shown significant tumor growth inhibition in αvβ3-positive glioblastoma models [97,98,99,100]. ^177^Lu-DOTA-E(cRGDfK)2 has demonstrated significant anti-tumor effect, indicating its potential for peptide receptor radionuclide therapy [101]. Similar anti-tumor effects were reported in melanoma using a ^177^Lu-PAMAM-DOTA-cRGDfK counterpart [102]. In preclinical models of pancreatic ductal adenocarcinoma expressing both αvβ3 and αvβ6, ^225^Ac-DOTA-RGD2 effectively inhibits tumor growth and extends survival with minimal toxicity, supporting the potential of the development of targeted alpha-therapy [103].

Despite promising preclinical results, clinical research on RGD-radiotracers for cancer therapy remains limited. ^177^Lu-DOTA-RGD2 was evaluated in a patient with thyroid cancer based on TEP images obtained after ^68^Ga-DOTA-RGD2 injection. Post-therapy imaging demonstrated reduced tracer uptake and significant clinical improvement [104]. This represents the first reported use of ^68^Ga-DOTA-RGD2 and ^177^Lu-DOTA-RGD2 in theranostic treatment, paving the way for future studies. Additionally, an ongoing clinical trial is assessing the safety and dosimetry of ^177^Lu-AB-3PRGD2 in lung cancer patients, with results expected by late 2024 [105].

These advancements highlight the promise of RGD-based nanoparticles and radiopharmaceuticals in overcoming cancer therapy challenges, enhancing drug delivery and efficacy while reducing side effects. However, issues related to tumor microenvironment complexity and targeting precision persist, indicating the need for further refinement and optimization of this targeting strategy.

### 3.3. Challenges and Limitations in Integrin Targeting

Integrin-targeted therapies, particularly those utilizing RGD peptides such as cilengitide, have shown substantial promise in preclinical cancer models, including osteosarcoma, medulloblastoma, and glioblastoma [39,84,106]. However, their translation into clinical success has faced numerous challenges. One major issue is the variability in integrin expression across different tumor types and even within different regions of the same tumor [9]. Tumor heterogeneity can result in inconsistent therapeutic outcomes. Some tumor cells may not express the targeted integrins or their ligands, or they may express them at insufficient levels for effective targeting. This heterogeneity is widely recognized as a major factor contributing to therapeutic resistance and treatment failure [107]. By understanding the underlying drivers of this heterogeneity, we could develop novel strategies to overcome treatment resistance.

Another significant challenge is the redundancy of integrin functions, which complicates targeting strategies [22]. Since multiple integrins can bind to the same extracellular matrix (ECM) proteins, blocking a single integrin might not be enough to disrupt tumor growth or metastasis, as other integrins could compensate for its loss. This redundancy necessitates designing therapies that can effectively target multiple integrins or pathways to achieve meaningful therapeutic effects. Additionally, integrins may have context-dependent roles; for example, some may promote tumor growth in primary tumors while inhibiting metastasis, or vice versa [108].

This dynamic behavior of integrins across cancer stages complicates drug development, as integrin expression can change within tumors over time. This makes it difficult to identify consistent therapeutic targets, as a drug effective in early-stage cancer may not work in later stages [109].

Moreover, the sequestration of RGD-targeted drugs by tumor-derived extracellular vesicles (TEVs) presents another obstacle. TEVs, which are prevalent in the tumor microenvironment, may express the same integrins as the tumor cells, leading to the inadvertent absorption of therapeutic agents before they reach their intended targets [57,59,110]. This sequestration reduces the efficacy of the treatments and poses a significant challenge to RGD-targeted therapies.

**Figure 1:**
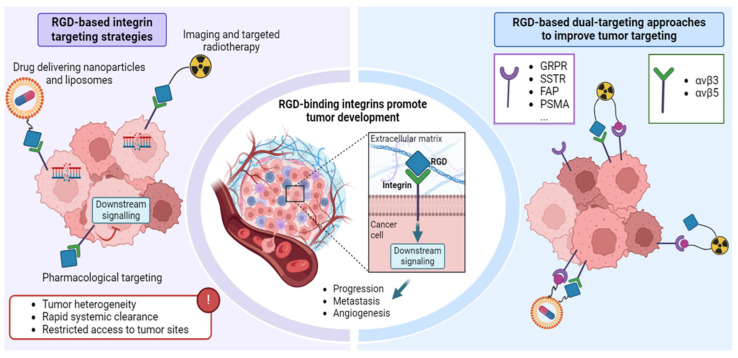
Schematic representation of tumors with high RGD-binding integrin expression, which drives tumor progression, metastasis, and angiogenesis. The figure illustrates various strategies targeting RGD-binding integrins, such as drug delivery through nanoparticles and liposomes, imaging and radiotherapy with radiopharmaceuticals, and pharmacological intervention using RGD inhibitors. Additionally, dual-targeting approaches are shown, which combine the targeting of RGD-binding integrins with other molecular targets. These dual-targeting strategies aim to improve the specificity and efficacy of both diagnostic and therapeutic interventions by simultaneously addressing multiple pathways involved in tumor growth and resistance, offering enhanced therapeutic outcomes compared to traditional single-target approaches.

Given these challenges, a promising strategy is to employ dual targeting approaches that combine RGD with other targeting moieties or therapeutic strategies. This dual approach could help overcome issues related to integrin redundancy and variability by ensuring that a broader spectrum of tumor cells is targeted more effectively, while also reducing the likelihood of therapeutic agents being sequestered by TEVs. In this way, double targeting could significantly improve the outcomes of cancer management with RGD-based therapies, making them more effective in clinical settings (Figure 1).

## 4. Dual-Targeting Approaches Based on RGD Peptides

Heterobivalent ligands provide notable advantages over monovalent agents in cancer management by targeting multiple receptors simultaneously or independently. This approach is particularly advantageous for tumors characterized by heterogeneous receptor expression, where single-target strategies may fall short [17]. Monospecific agents, such as RGD-based radiotracers, have shown potential, and their efficacy can be limited by partial receptor expression, resulting in incomplete lesion detection. While the use of separate monospecific agents could address this issue by targeting different receptors individually, this strategy presents several drawbacks, including increased regulatory hurdles, heightened patient discomfort, and elevated costs. In contrast, heterobivalent approaches, which use multifunctional platforms like radiotracers, nanoparticles, and liposomes, simplify the process with a single application, enhancing stability, avidity, and tumor detection [15]. In this review, we delve into RGD-based dual-targeting strategies, mostly focusing on radiotracers (Table 2) that target αvβ3 and other biomarkers to advance cancer diagnosis and treatment.

### 4.1. Dual-Targeting RGD-Based Radiotracers for Imaging and Targeted Therapy

#### 4.1.1. Dual Targeting of GRPR and αvβ3

Gastrin-releasing peptide receptor (GRPR) is a is a G protein-coupled receptor that binds to the neuropeptide gastrin-releasing peptide (GRP), playing a critical role in stimulating gastric acid secretion and regulating gastrointestinal motility [149]. GRPR contributes to tumor growth and progression and is overexpressed in various cancers, including prostate, breast, small cell lung (SCLC), colon, gastrointestinal, and pancreatic cancer, as well as glioblastomas and neuroblastomas [150]. Due to its expression pattern, GRPR is an attractive target for cancer imaging and therapy. Theranostic agents targeting GRPR are currently under clinical investigation [151].

One dual-targeting tracer developed for GRPR and αvβ3 was developed, the BBN-RGD. After successful preclinical studies in mouse models of pancreatic tumors, which demonstrated higher tumor uptake of ^68^Ga-BBN-RGD compared to ^68^Ga-BBN or ^68^Ga-RGD alone, a first-in-human trial evaluated the safety and diagnostic effectiveness of ^68^Ga-NOTA-BBN-RGD in prostate cancer patients [111,112]. The study showed no adverse effects and confirmed the radiotracer’s safety, with a radiation dose well below FDA limits. ^68^Ga-NOTA-BBN-RGD PET/CT detected more primary tumors (3/4 vs. 2/4 for ^68^Ga-BBN), metastatic lymph nodes (14 vs. 5), and bone lesions (20 vs. 12) compared to ^68^Ga-BBN alone, indicating superior diagnostic performance. This result prompted additional evaluation of the radiotracer in breast cancer patients [113]. The findings revealed substantial ^68^Ga-NOTA-BBN-RGD accumulation in primary tumors and metastases, correlating with GRPR and αvβ3 expression. In 11 patients who underwent two PET scans in this study, ^68^Ga-BBN-RGD PET/CT identified 13 suspected primary tumor lesions in 9 patients, along with 8 metastatic lymph nodes, 9 bone metastases, and 4 lung metastases. In comparison, ^68^Ga-BBN PET/CT detected 11 suspected primary tumor lesions in 7 patients, along with 3 metastatic lymph nodes, 3 bone metastases, and 2 lung metastases.

Another dual-targeting tracer, RGD-Glu-[DO3A]-6-Ahx-RM2, was developed by linking RGD and RM2 peptides with a DOTA chelating, radiolabeled with ^86^Y or ^90^Y and tested in preclinical models [114]. Biodistribution studies in mice bearing pancreatic tumors revealed significant tumor uptake and retention of the tracer. Micro PET imaging supported these findings, showing effective targeting and retention, which positions RGD-Glu-[DO3A]-6-Ahx-RM2 as a promising candidate for further imaging studies in larger animal models and potentially in clinical settings.

Finally, the recently developed radiotracer RM26-RGD (LNC1015) has shown strong potential as a dual-targeting agent for GRPR and αvβ3 in PET imaging [115]. In preclinical studies using a pancreatic xenograft model, ^68^Ga-RM26-RGD demonstrated high stability, favorable binding affinity, and superior tumor uptake compared to the monomeric tracers ^68^Ga-RGD and ^68^Ga-RM26. Early clinical trials in breast cancer patients corroborated these findings, revealing significantly higher tumor uptake and improved tumor-to-background ratios compared to ^18^F-FDG. Additionally, in a cohort of 23 brain tumor patients, ^68^Ga-RM26-RGD demonstrated high tumor uptake and favorable tumor-to-background ratios compared to the monomeric tracers ^68^Ga-RM26 and ^68^Ga-BBN, with a moderate positive correlation with tumor grade [116]. Currently, this tracer is being further evaluated in a clinical trial for PET/CT imaging of breast, brain, and prostate cancers, where its diagnostic efficacy is being compared to ^18^F-FDG, ^68^Ga-RGD, and ^68^Ga-RM26 (NCT05549024). Overall, these findings highlight the potential of dual-targeting radiotracers that focus on αvβ3 integrin and GRPR to improve the accuracy of cancer diagnosis and staging in specific cancer types.

#### 4.1.2. Dual Targeting of SSTR and αvβ3

Somatostatin receptors (SSTRs) are a class of G protein-coupled receptors that bind somatostatin, a peptide hormone that inhibits the release of several other hormones and regulates various physiological [152]. There are five known subtypes of SSTRs (SSTR1-5), which are expressed in various tissues including the brain, gastrointestinal tract, and pancreas. SSTRs are overexpressed in many neuroendocrine tumors, making them critical targets for both diagnostic imaging and therapy [153]. Radiolabelled somatostatin analogs, such as ^68^Ga-DOTATATE and ^177^Lu-DOTATATE (Luthatera^®^), are commonly used in PET imaging and targeted radionuclide therapy, respectively, to detect and treat SSTR-expressing tumors. Lutathera^®^ became the first FDA-approved peptide receptor radionuclide therapy (PRRT) following the NETTER-1 trial, which demonstrated a remarkable progression-free survival (PFS) in comparison to octreotide [154]. Despite these advances, resistance to PRRT can develop, necessitating the exploration of alternative therapeutic strategies, such as dual-targeting approaches.

The radiotracer ^68^Ga-NOTA-3P-TATE-RGD has been recently evaluated for its dual-targeting properties towards SSTR2 and αvβ3. This tracer exhibited binding affinities comparable to its monomeric counterparts in both in vitro and in vivo models of small (SCLC) and non-small cell lung cancers (NSCLC), suggesting its broad applicability for detecting cancers involving both SSTR2 and integrin αvβ3 [117]. In a proof-of-concept study involving 32 patients with NSCLC and SCLC, ^68^Ga-NOTA-3P-TATE-RGD provided high-quality imaging with significantly improved tumor-to-background (T/B) ratios compared to its monomeric forms [118]. Immunohistochemical analysis further confirmed the presence of SSTR2 and variable integrin αvβ3 levels in tumor lesions, highlighting the tracer’s effectiveness in dual targeting.

In a subsequent study involving 35 patients with neuroendocrine tumors (NETs), ^68^Ga-NOTA-3P-TATE-RGD identified more liver lesions (634 vs. 532, *p* = 0.021) and showed superior T/B ratios in the liver compared to ^68^Ga-DOTATATE (8.4 ± 5.5 vs. 4.7 ± 3.7, *p* < 0.001), proving particularly effective in detecting FDG-avid NETs [119]. Finally, in a cohort of 12 patients with radioiodine-refractory thyroid carcinoma (RAIR-TC)—a type of thyroid cancer resistant to traditional iodine-based treatments—^68^Ga-NOTA-3P-TATE-RGD demonstrated slightly higher T/B ratios for lymph node metastases and was comparable to ^18^F-FDG in detecting other metastases [120,155]. Overall, ^68^Ga-NOTA-3P-TATE-RGD emerges as a versatile dual-targeting radiotracer that enhances imaging and detection across various cancers, improving diagnostic precision and therapeutic management.

#### 4.1.3. Dual Targeting of FAP and αvβ3

Fibroblast activation protein (FAP) is a serine protease predominantly expressed in the stroma of solid tumors including breast, pancreatic, and colorectal cancers, while being minimally present in normal tissues [156]. Its elevated expression correlates with tumor progression and poor prognosis, making it a promising target for imaging and therapy. FAP is commonly targeted with radiotracers such as Fibroblast Activation Protein Inhibitors (FAPIs), which specifically bind to FAP, enabling precise imaging and potential therapeutic intervention in tumor-associated stromal cells [157,158,159].

The dual-targeting PET tracer ^68^Ga-NOTA-FAPI-RGD, designed to target both FAP and integrin αvβ3, demonstrated significantly enhanced tumor uptake, retention, and TBR compared to its monomeric counterparts ^68^Ga-FAPI-02 and ^68^Ga-RGDfK in preclinical pancreatic tumor models [121]. Indeed, tumor uptake of ^68^Ga-NOTA-FAPI-RGD was 5.33 ± 0.27% ID/g, compared to 2.89 ± 0.09% ID/g for ^68^Ga-RGDfK and 1.16 ± 0.07% ID/g for ^68^Ga-FAPI-02 at 2 h post-injection. This result led to a preliminary clinical study involving six newly diagnosed cancer patients, where ^68^Ga-NOTA-FAPI-RGD showed favorable pharmacokinetics with rapid and high tumor uptake, prolonged retention, effective dosimetry and comparable maximal uptake to ^18^F-FDG, underscoring its strong diagnostic potential [121].

In a pilot study involving 51 patients with suspected lung malignancies, ^68^Ga-NOTA-FAPI-RGD outperformed ^18^F-FDG, ^68^Ga-RGD, and ^68^Ga-FAPI-04 in detecting primary tumors and metastases, and exhibited superior accuracy in evaluating mediastinal lymph nodes [122]. Further clinical evaluation in 22 patients across various cancer types revealed that ^68^Ga-FAPI-RGD had higher tumor uptake and TBR compared to ^18^F-FDG and ^68^Ga-FAPI-46, leading to an improved detection of primary tumors, lymph node, and bone metastases. This study also evaluated the safety and effectiveness of the tracer [123]. The enhanced imaging capability of ^68^Ga-NOTA-FAPI-RGD was attributed to its dual targeting of FAP and integrin αvβ3, making it more effective in detecting both tumor and stromal components.

In comparative studies, ^68^Ga-NOTA-FAPI-RGD (LNC1007) exhibited superior detection of primary tumors and metastases in 61 patients across different cancers compared to ^18^F-FDG and ^68^Ga-FAPI-02 PET/CT [124]. It also showed enhanced performance in renal cell carcinoma (RCC) imaging, surpassing ^18^F-FDG and ^68^Ga-PSMA in detecting primary lesions, skeletal, and peritoneal metastasis, with strong correlations to FAP expression [125]. Additionally, the tracer effectively distinguishes between aggressive and less aggressive RCC. Finally, a recent case report showed ^68^Ga-NOTA-FAPI-RGD successfully identifying metastasis in a case of radioiodine-refractory thyroid cancer, with superior uptake and clearer lesion delineation compared to ^18^F-FDG, highlighting its potential for targeted treatment [126].

A comparison of ^68^Ga- and ^18^F-AlF-LNC1007 revealed similar pharmacokinetics, with both tracers showing high tumor uptake and prolonged retention in preclinical glioblastoma models [127]. Clinical evaluations of ^18^F-AlF-LNC1007 in six cancer patients confirmed its stability, effective imaging performance, and increasing TBR over time. In a study involving 33 patients with breast cancer, ^18^F-AlF-LNC1007 was compared with ^18^F-FDG and ^18^F-FAPI-04. It showed higher uptake in primary tumors and metastases than ^18^F-FDG but showed lower TBR for bone metastases [128]. In contrast, ^18^F-FAPI-04 had better performance in maximal uptake for all lesions, including bone metastases, while ^18^F-FDG excelled in imaging liver metastases and correlated well with metastatic tumor volume in small bone lesions. A clinical trial is currently recruiting to evaluate the pharmacokinetics, biodistribution, dosimetry, and safety of ^18^F-LNC1007 in healthy volunteers and cancer patients with minimal tumor burden (NCT06471712).

While FAPI-RGD-based tracers have demonstrated exceptional performance in clinical diagnostics, ongoing research aims to optimize their structure for enhanced efficacy in targeted radiotherapy. The DOTA-FAPI-RGD tracer, labeled with either ^68^Ga or ^177^Lu, has shown high yields, stability, strong binding, and rapid internalization in FAP/αvβ3-positive glioblastoma cells [129]. In preclinical models, this tracer achieved high tumor uptake, rapid clearance, and clear imaging. The ^177^Lu-labeled version of this heterodimeric ligand shows promise for therapeutic applications. Preclinical studies involving ^68^Ga- or ^177^Lu-labeled FAP-RGD, synthesized from FAP-2286 and c(RGDfK), demonstrated strong binding affinity, increased tumor uptake, and prolonged retention compared to their monomeric counterparts [130]. These studies confirm the tracer’s favorable imaging properties and effective antitumor responses, highlighting its potential for targeted radiotherapy.

A recent work of Wen et al., highlighted the potential of ^177^Lu-DOTA-EB-FAPI-RGD (LNC1009), a modification of LNC1007 with an Evans Blue motility to enhance tumor targeting, showed great promise for the imaging and therapy of solid tumors that are FAP^+^/α_v_β_3_^-^, FAP/α_v_β_3_^+^, or FAP^+^/α_v_β_3_^+^ [131].

The promising results from dual-targeting PET tracers like ^68^Ga-FAPI-RGD, ^18^F-AlF-LNC1007, or ^177^Lu-LNC1009 highlight their potential to significantly improve both diagnostic accuracy and therapeutic outcomes by enhancing tumor visualization and targeting capabilities, particularly in complex cancer cases. However, further investigations are warranted to determine their clinical value.

#### 4.1.4. Dual Targeting of PSMA and αvβ3

This dual-target strategy also takes advantage of the recent development of PSMA-derived tracers. PSMA is a glycoprotein predominantly expressed on the surface of prostate epithelial cells and is significantly overexpressed in prostate cancer, particularly in advanced and metastatic forms [160]. PSMA contributes to cancer progression by promoting tumor cell growth, invasion, and angiogenesis, and it interacts with the extracellular matrix, which facilitates metastasis [161,162]. Its limited expression in normal tissues makes PSMA an ideal target for both diagnostic imaging and targeted therapies, improving the precision and effectiveness of prostate cancer management [163,164,165].

As heterobivalent peptides gain prominence in targeted radionuclide therapy, the ^177^Lu-iPSMA-RGD peptide, which targets both PSMA and αvβ3 integrins, serves as a key example, designed to enhance tumor targeting and improve therapeutic results. The peptide demonstrated a strong binding affinity to PSMA and αvβ3 integrins in vitro, as well as an effective reduction in cell viability and induced apoptosis in U87MG glioblastoma cells [132]. While iPSMA-RGD showed potential for dual targeting, it did not outperform the individual components in receptor recognition, warranting further preclinical studies to assess its therapeutic potential.

For colorectal cancer, ^225^Ac-iPSMA-RGD showed increased cytotoxicity in HCT116 cells, inducing significant DNA damage, apoptosis, and cell death [133]. In mice, tumor growth was markedly reduced with ^225^Ac-iPSMA-RGD compared to untreated controls. Eleven days after treatment, ^225^Ac-iPSMA-RGD achieved a greater tumor reduction than ^225^Ac-RGD and ^225^Ac-PSMA. These findings, reflecting the ablative doses of the radiopharmaceuticals, suggest that ^225^Ac-iPSMA-RGD has strong potential for treating colorectal cancer, similar to the effectiveness seen with higher doses in metastatic prostate cancer. The clinical applicability of such treatments needs to be further investigated.

#### 4.1.5. Dual Targeting of APN/CD13 and αvβ3

Aminopeptidase N (APN), also known as CD13, is a cell surface metalloprotease involved in various physiological and pathological processes, including tumor growth and angiogenesis [166]. It is highly expressed on the surface of tumor-associated endothelial cells and certain cancer cells, making it a valuable target for cancer imaging and therapy. APN interacts indirectly with integrins by influencing the extracellular matrix and modulating cell adhesion and migration processes, which are crucial for tumor progression [167].

The dual-targeting PET imaging tracer ^68^Ga-NGR-RGD was designed to target both integrin αvβ3 and APN (CD13) [134]. In breast cancer models, this tracer demonstrated high stability and specificity, achieving superior tumor uptake and contrast compared to single-target tracers and detecting metastatic lung lesions. Further evaluation in ovarian cancer models showed that ^68^Ga-NGR-RGD provided better contrast and more clearly delineated peritoneal and liver metastases than ^18^F-FDG [135]. Renamed ^68^Ga-HX01, it was tested in various tumor models, including those for pancreas, breast, gallbladder, lung, ovary, colorectal, liver, stomach, and glioma cancers, outperforming single-target probes and ^18^F-FDG with higher tumor uptake and improved tumor-to-background ratios [136].

The promising results led to the initiation of a clinical translation. A Phase Ia study was conducted to evaluate the safety, biodistribution, radiation dose, and pharmacokinetics of ^68^Ga-HX01 in healthy Chinese adults, while a Phase Ib study focused on its safety and tumor imaging capabilities in patients with malignant solid tumors (NCT06416774). Despite these advances, ^68^Ga-HX01 seems to face challenges with fast clearance and limited tumor retention. To address these issues, a new radiopharmaceutical, ^68^Ga-HX01-L6, was developed by adding an albumin binder to the HX01 structure [137]. ^68^Ga-HX01-L6 demonstrated significantly improved tumor uptake and retention in pancreas xenograft models. When labeled with ^177^Lu, it showed rapid clearance from normal tissues and high tumor uptake, positioning it as a promising candidate for therapeutic applications. Further studies are needed to fully evaluate its therapeutic potential and confirm its role in clinical settings.

#### 4.1.6. Dual Targeting of NRP-1 and αvβ3

Neuropilin-1 (NRP-1) is a cell surface receptor that plays a crucial role in cell signaling, angiogenesis, and tumor progression [168]. Overexpressed in several cancers, NRP-1 enhances tumor growth by promoting angiogenesis and cell migration, often in conjunction with vascular endothelial growth factor (VEGF) [169]. Its interaction with these factors makes NRP-1 a valuable target for imaging and therapies aimed at disrupting tumor vasculature and limiting cancer spread [168,170,171].

A radiolabeled heterodimeric peptide, ^18^F-AlF-NOTA-RGD-ATWLPPR, was designed to target both integrin αvβ3 and NRP-1 in tumors [138]. This peptide showed enhanced tumor uptake and targeting compared to monomeric RGD and ATWLPPR (A7R). In vitro and in vivo studies using GBM tumor models showed that the heterodimer’s uptake was only partially inhibited by an excess of either unlabeled RGD or ATWLPPR, but it was completely blocked when both were present, leading to improved pharmacokinetics and imaging quality.

In another study, a tetrameric RGD-A7R peptide conjugate labeled with a fluorescent probe exhibited superior tumor uptake, imaging contrast, and pharmacokinetics compared to monomeric peptides [172].

A recent study introduced the heterodimeric tracer ^68^Ga-DOTA-RGD-A7R, which targets both αvβ3 and NRP-1 [139]. In breast cancer xenograft mouse models, this tracer showed excellent stability, clear tumor imaging on PET/CT, and superior tumor uptake compared to its individual components. Blocking studies validated the specificity of ^68^Ga-DOTA-RGD-ATWLPPR for its intended targets [139,140]. These studies highlight the potential of dual-targeting tracers, such as ^18^F-AlF-NOTA-RGD-ATWLPPR and ^68^Ga-DOTA-RGD-ATWLPPR, in enhancing the precision of cancer imaging and therapy by effectively targeting both integrin αvβ3 and NRP-1, offering new avenues for advancing cancer diagnosis and treatment. However, no clinical studies have been conducted until today.

#### 4.1.7. Dual Targeting of Growth Factors Receptors (GFRs) and αvβ3

Growth factor receptors are well-established targets in oncology, with dual-targeting agents incorporating RGD peptides developed to enhance the efficacy of their inhibitors. Among them, studies have focused on Vascular endothelial growth factor receptor (VEGFR). VEGFR promotes tumor growth by facilitating new blood vessel formation, while integrin αvβ3, often co-expressed with VEGFR, helps stabilize these vessels [173]. Radiotracers targeting both VEGFR and integrin αvβ3 provide detailed imaging of the tumor’s blood supply and active regions.

A notable example is the development of a peptide targeting VRGFR and αvβ3, iRGD-C6-lys-C6-DA7R radiolabelled with ^211^At, investigated recently for targeted radiotherapy [141]. The peptide demonstrated stability, strong binding to U87MG glioma cells, and significant in vitro antitumor effects, including reduced cell viability and increased apoptosis. Biodistribution studies revealed rapid tumor uptake and clearance from normal tissues via the kidneys. In vivo, it effectively inhibited tumor growth and extended survival in glioma-bearing mice with minimal toxicity. These results suggest that VEGFR targeting alongside αvβ3 is a promising strategy for detecting and treating glioma and potentially other cancers

Similarly, epidermal growth factor receptor (EGFR), another crucial actor of tumor cell growth and survival, was targeted by such an approach. Radiotracers that target EGFR alongside integrin αvβ3 offer insights into both the tumor cells and their vascular support, providing a more comprehensive view of tumor biology. This dual-targeting approach could bolster diagnostic accuracy and treatment planning by integrating multiple aspects of tumor dynamics.

Innovative heterodimeric peptides have been introduced, such as ^68^Ga-NOTA-RGD-GE11, which combine integrin αvβ3-specific cyclic RGD peptides with the EGFR-targeting peptide GE11 [142]. This dual targeting showed significantly improved tumor uptake compared to its monomeric counterparts, ^68^Ga-NOTA-RGD and ^68^Ga-NOTA-GE11, in lung tumor models. At 2 h post-injection, the tumor uptake values for ^68^Ga-NOTA-RGD-GE11, ^68^Ga-NOTA-RGD, and ^68^Ga-NOTA-GE11 were 3.446 ± 0.548, 2.756 ± 0.483, and 2.408 ± 0.327% ID/g. Similarly, the ^64^Cu-labeled tracer, ^64^Cu-NOTA-RGD-GE11, exhibited superior tumor uptake in pancreatic xenografts and showed enhanced specificity, with uptake significantly blocked by non-radioactive peptides [143]. Additional development of NODA-GA-PEG3-GE11-PEG3-c(RGDyK) and c(RGDfK) tracers highlighted the feasibility of creating radiolabelled heterobivalent peptides (HBPLs) [144]. Although these HBPLs were successfully labeled with ^68^Ga, exhibited high hydrophilicity, and demonstrated stability, they showed strong binding to integrin αvβ3 but lacked specific interaction with EGFR in vitro. In vivo studies in squamous cell carcinoma models have confirmed that uptake was mediated exclusively by integrin αvβ3. Given the mixed results of the dual-targeting approach with GE11, future research should explore alternative EGFR-specific peptides to enhance dual-targeting effectiveness and improve tumor imaging.

#### 4.1.8. Dual Targeting of uPAR and αvβ3

uPAR is a cell surface receptor crucial for cell migration, tissue remodeling, and inflammation. uPAR is frequently overexpressed in cancers such as breast, prostate, and lung cancer, where it is associated with increased tumor invasion and poor prognosis [174]. This makes uPAR a valuable biomarker for imaging and monitoring tumor activity through PET and SPECT radiotracers.

In a proof-of-concept study, researchers developed heterodimeric ligands targeting both uPAR and integrin αvβ3 using an innovative bifunctional chelator (BFC) scaffold [145]. These ligands combined the peptide ligands AE105 (for uPAR) and cyclo(RGDyK) and were radiolabelled with ^64^Cu and ^68^Ga. The heterodimers demonstrated enhanced stability and superior imaging performance compared to single-target radiotracers. PET imaging of U87MG tumor-bearing mice with the ^64^Cu-labeled tracer showed improved tumor targeting capabilities, with higher tumor uptake and clearer images than those achieved with individual-target tracers.

Subsequent studies further optimized these heterodimeric probes [146]. The tracers continued to exhibit better binding affinity and higher receptor density than their monomeric counterparts. Among the tested variants, the heterodimer with a PEG8 linker delivered the best results, showing strong tumor retention and stability in both in vitro and in vivo assays when labeled with ^64^Cu and ^68^Ga [146,147]. PET imaging of U87MG and PANC-1 tumor xenografts demonstrated that this PEG8-linked heterodimer significantly outperformed monomeric tracers, achieving higher tumor uptake and a superior signal-to-background ratio, highlighting its potential as a highly effective imaging agent for tumors expressing both uPAR and integrin αvβ3.

#### 4.1.9. Dual Targeting of MC1R and αvβ3

This dual-targeting approach was also recently evaluated for melanoma imaging and targeting. Melanocortin-1 receptor (MC1R) is a G protein-coupled receptor primarily expressed in melanocytes, where it regulates melanin production. Activation of MC1R stimulates the synthesis of eumelanin, providing photoprotection to the skin. MC1R is critical in melanoma, with its variants increasing UV sensitivity and cancer risk. In tumors, MC1R affects immune response, tumor growth, and metastasis by influencing cellular adhesion and migration [175,176]. Its dysregulation can facilitate melanoma progression and spread.

A recent study focused on developing a heterobivalent radiotracer targeting both the MC1R and integrin αvβ3, which play critical roles in melanoma progression and metastasis [148]. Six ligands, incorporating c(RGDfK) and GG-Nle-c(DHfRWK) (Gly-Gly-Nle-cyclic Asp-His-DPhe-Arg-Trp-Lys), were synthesized and radiolabelled with ^18^F to enhance tumor-targeting sensitivity and visualization. Among them, one compound (^18^F-SiFAlin-GG-Nle-c(DHfRWK)-PEG8-RGD) demonstrated strong receptor affinity, hydrophilicity, and superior tumor uptake and TBR in melanoma and glioblastoma mouse models. This makes it a promising radiotracer for PET/CT imaging of malignant melanoma. However, studies in patients should be performed to fully characterize the potential of such radiotracer.

### 4.2. Dual-Targeting RGD-Based Nanoparticles and Liposomes; Implications for Brain Tumor Management

In addition to major recent developments in the theranostic field, advancements in targeted delivery systems have emerged, particularly for glioma therapy. Several innovations rely on RGD-derived peptides to enhance both tumor specificity and blood–brain barrier (BBB) penetration. Polyamidoamine (PAMAM) dendrimers, known for their stability and nanoscale size, are optimized for glioma therapy through PEGylation, which promotes passive targeting via the Enhanced Permeability and Retention (EPR) effect. Further, RGDyC modification provides active targeting by binding to integrin αvβ3 receptors on tumor cells [177]. In a rat glioma model, the RGDyC-modified PEG-PAMAM/ATO (arsenic trioxide) system showed superior therapeutic efficacy and significant anti-tumor effects as compared to ATO alone and mPEG-PAMAM/ATO due to improved BBB penetration and targeted delivery [178]. RGDyC-modified PEG-PAMAM/ATO was also associated with reduced secondary effects in this model. However, despite these combined passive and active targeting strategies, effectively delivering therapeutics across the BBB remains a challenge.

To address this issue, PAMAM was further modified with both the iRGD peptide for tumor-specific targeting via integrin αvβ3 and the TGN peptide, which binds to transferrin receptors on BBB endothelial cells to enhance brain penetration [179]. The resulting system (iRGD/TGN-PEG-PAMAM) loaded with ATO showed high entrapment efficiency, pH-sensitive release, and significantly improved drug delivery across the BBB. In vitro and in vivo studies have confirmed an enhanced accumulation and activation of ATO in glioma tissue, leading to increased therapeutic efficacy and reduced side effects. This dual-targeting approach demonstrates a promising strategy for targeted glioma therapy by optimizing both tumor specificity and BBB penetration.

Building on these advancements, a hepatitis B core protein-based virus-like particle (TGN/RGD-VLP) was developed to co-deliver paclitaxel (PTX) and siRNA to glioblastoma models [180]. This approach effectively targeted both the αvβ3 integrin and TGN, resulting in enhanced drug delivery to tumor sites. The combined therapy (PTX/siRNA@TGN/RGD-VLP) outperformed PTX alone, significantly reducing tumor growth, promoting necrosis and apoptosis, extending median survival, and minimizing weight loss.

Further advancing targeted delivery for glioma therapy, a study developed chitosan-PLGA nanoparticles functionalized with AS1411 aptamer, targeting nucleolin overexpressed on brain cancer cells, and RGD peptide, to enhance the targeted co-delivery of docetaxel (DTX) and up conversion nanoparticles (UCNP) for brain cancer therapy and imaging [181]. These nanoparticles, with an average size of less than 200 nm, exhibited high DTX and UCNP encapsulation and sustained DTX release over 72 h. The dual-functional nanoparticles significantly improved cellular uptake and cytotoxicity, showing an 89-fold greater efficacy than unmodified particles. Additionally, they increased DTX bioavailability and UCNP accumulation in brain tissues, leading to substantial tumor growth suppression in brain tumor-bearing mice without notable toxicity. The DUCPN-RGD-AS1411 nanoparticles demonstrate potential for effective brain cancer treatment and real-time imaging.

In a related development, a novel approach utilized dual-target liposomes to enhance drug delivery across the BBB. These liposomes were modified with a glucose-RGD (Glu-RGD) derivative to target glioma cells and facilitate BBB penetration [182]. The Glu-RGD-modified liposomes, encapsulating PTX, showed significantly improved targeting efficiency and accumulation at tumor sites compared to unmodified PTX and other liposome formulations. The liposomes demonstrated enhanced drug delivery with a 4.41-fold increase in uptake efficiency and a 4.72-fold increase in concentration efficiency at tumor sites. This method further underscores the progress in developing effective delivery systems that combine BBB penetration with targeted therapy for gliomas.

These advancements underscore the ongoing efforts to overcome the challenges of blood–brain barrier penetration and dual tumor targeting, paving the way for future innovations in brain cancer management. Future challenges will include the variability of the blood–brain barrier’s permeability among patients and potential differences in tumor biology between animal models and human cancers. Additionally, the long-term safety and effectiveness of these nanoparticle-based therapies need thorough evaluation. To confirm their potential, comprehensive clinical studies are necessary to assess their performance in human subjects, ensuring that these innovations can be safely and effectively applied in clinical settings.

## 5. Conclusions

Despite their promise, RGD-derived molecules designed for the treatment of solid tumors have faced several challenges, including rapid clearance, potential immunogenicity, and heterogeneity in integrin expression. Dual-targeting strategies based on RGD in cancer imaging and therapy have made notable progress, using radiotracers to target multiple biomarkers or receptors, which could enhance both diagnostic precision and therapeutic efficacy. Additionally, recent advancements in targeted delivery systems, such as RGD-based nanoparticles and liposomes, have improved drug delivery across the blood–brain barrier (BBB) and boosted therapeutic effectiveness, highlighting the need for continued development. However, challenges persist, including variability in BBB permeability and differences between animal models and human cancers. Future research should focus on refining dual-targeting approaches by investigating specific mechanisms that could enhance tumor selectivity while minimizing off-target effects. For many dual-targeting strategies, the therapeutic efficacy has yet to be fully validated in preclinical models, where studies on biodistribution and pharmacodynamics could offer valuable insights into their potential effectiveness. Additionally, it is essential to optimize these systems to balance potency and safety. Finally, comprehensive clinical trials are needed to assess not only the safety and efficacy of these innovations but also their long-term immunogenicity, patient-specific responses, and potential for personalized treatment adaptations. These trials will be crucial for translating dual-targeting therapies from experimental settings to widespread clinical use.

In conclusion, dual-targeting strategies and advanced delivery systems could lead to significant progress in cancer management, promising better diagnosis, treatment precision, and improved patient outcomes. Clinical trials involving RGD-based dual molecules are crucial to establishing their role in cancer therapy.

## Figures and Tables

**Table 1:** RGD-derived radiotracers recently evaluated in humans for the non-invasive imaging of integrins.

Radiotracers	Application	Tumor Type	Clinical Trial	References
^18^F-Galacto-RGD	PET imaging	GBM, HNSCC, prostate cancer	Phase I	[64,65,66]
^18^F-Alfatide	PET imaging	Lung cancer, GBM	Phase I	[67,68,69,70]
^68^Ga-NOTA-PRGD2	PET imaging	Lung cancer, gliomas	Phase I	[71,72,73]
^68^Ga-DOTA-RGD2	PET imaging	HSNCC, breast, thyroid cancer, and chondrosarcoma	Phase I and Case report	[74,75,76]
^68^Ga-NODAGA-E[c(RGDyK)]2	PET imaging	Breast, neuroendocrine tumors, and thyroid cancers	Phases I and II	[77,78,79]
^99m^Tc-3PRGD2	SPECT imaging	Lung cancer	Phase III	NCT04233476
^68^Ga-cycratide	PET imaging	Pancreatic cancer	First in human	[80]
^18^F-αvβ6-BP	PET imaging	Pancreatic cancer	First in human	[81]
^18^F-FP-R01-MG-F2	PET imaging	Pancreatic cancer	Phase I	[82]

**Table 2:** Dual-targeting heterobivalent radiotracers incorporating RGD motifs for tumor imaging and therapy currently in development.

Targets	RGD-Based Molecule	Radioisotope	Application	Tumor Model (In Vivo)	Clinical Trial	References
GRPR and αvβ3	NOTA-BBN-RGD	^68^Ga	PET imaging	PC3 mice	Prostate (First in human(FIH)) and breast cancer (Phase I)	[111,112,113], NCT02749019
	RGD-Glu-[DO3A]-6-Ahx-RM2	^86^Y/^90^Y	PET imaging and therapy	PC3 mice	/	[114]
	RM26-RGD (LNC1015)	^68^Ga	PET imaging	PC3 mice	Breast (FIH), brain (Phase I), and prostate cancer (Phase I)	[115,116], NCT05549024
SSTR and αvβ3	NOTA-3P-TATE-RGD	^68^Ga	PET imaging	H69 and A549 mice	SCLC and NSCLC (Phase I), GEP-NETs (Phase I), and RAIR-TC (Phase I)	[117,118,119,120]
FAP and αvβ3	FAPI-RGD (LNC1007)	^68^Ga	PET imaging	Panc02 mice	Various solid tumors (Phases I/II)	[121,122,123,124,125,126]
	AlF-LNC1007	^18^F	PET imaging	U87MG mice	Breast cancer (Phase I)	[127,128], NCT06471712
	DOTA-FAPI-RGD	^68^Ga/^177^Lu	PET/SPECT imaging and therapy	U87MG mice	/	[129]
	FAP-RGD	^68^Ga/^177^Lu	PET/SPECT imaging and therapy	HT1080-FAP and U87MG mice	/	[130]
	DOTA-EB-FAPI-RGD (LNC1009)	^177^Lu	SPECT imaging and therapy	U87MG mice	/	[131]
PSMA and αvβ3	iPSMA-RGD	^177^Lu	SPECT imaging and therapy	/	/	[132]
		^225^Ac	Therapy	HCT116 mice	/	[133]
APN/CD13 and αvβ3	NGR-RGD (HX01)	^68^Ga	PET imaging	Various tumor models	Solid tumors (Phase I)	[134,135,136], NCT06416774
	HX01-L6	^68^Ga/^177^Lu	PET/SPECT imaging and therapy	BxPC-3 mice	/	[137]
NRP-1 and αvβ3	AlF-NOTA-RGD-ATWLPPR	^18^F	PET imaging	U87MG mice	/	[138]
	DOTA-RGD-A7R	^68^Ga	PET imaging	MCF-7 mice	/	[139,140]
VEGFR and αvβ3	iRGD-C6-lys-C6-DA7R	^211^At	Therapy	U87MG mice	/	[141]
EGFR and αvβ3	NOTA-RGD-GE11 NODA-GA-PEG3-GE11-PEG3-RGD	^68^Ga	PET imaging	NCI-H292 mice	/	[142]
		^64^Cu	PET imaging	BxPC3 mice	/	[143]
		^68^Ga	PET imaging	A431 mice	/	[144]
uPAR and αvβ3	AE105-NOTA-RGD	^68^Ga/^64^Cu	PET imaging	U87MG mice	/	[145]
	AE105-PEG8-NOTA-PEG4-RGD	^68^Ga/^64^Cu	PET imaging	U87MG and PANC-1 mice	/	[146,147]
MC1R and αvβ3	SiFAlin-GG-Nle-c(DHfRWK)-PEG8-RGD	^18^F	PET imaging	B16F1 mice	/	[148]
